# Spectroscopic Evaluation of the Potential Neurotoxic Effects of a New Candidate for Anti-Seizure Medication—TP-315 during Chronic Administration (In Vivo)

**DOI:** 10.3390/ijms23094607

**Published:** 2022-04-21

**Authors:** Mikolaj Krysa, Anna Makuch-Kocka, Katarzyna Susniak, Tomasz Plech, Marta Andres-Mach, Mirosław Zagaja, Anna Sroka-Bartnicka

**Affiliations:** 1 Independent Unit of Spectroscopy and Chemical Imaging, Faculty of Biomedical Sciences, Medical University of Lublin, Chodzki 4a, 20-093 Lublin, Poland; mikolajkrysa@umlub.pl; 2 Department of Pharmacology, Chair of Pharmacology and Biology, Faculty of Health Sciences, Medical University of Lublin, 20-093 Lublin, Poland; tomasz.plech@umlub.pl; 3 Department of Genetics and Microbiology, Institute of Biology and Biotechnology, Maria Curie-Sklodowska University, 20-033 Lublin, Poland; katarzyna.susniak@umlub.pl; 4 Chair and Department of Pharmaceutical Microbiology, Faculty of Pharmacy, Medical University of Lublin, 20-093 Lublin, Poland; 5 Isobolographic Analysis Laboratory, Institute of Rural Health, 20-090 Lublin, Poland; andres.marta@imw.lublin.pl (M.A.-M.); zagaja.miroslaw@imw.lublin.pl (M.Z.)

**Keywords:** epilepsy, anti-seizure medication, 1,2,4-triazole-3-thione derivatives, neurotoxicity, FT-IR imaging, spectroscopy

## Abstract

The aim of this study was to investigate the potential neurotoxic effect of the new anti-seizure medication candidate—5-(3-chlorophenyl)-4-hexyl-2,4-dihydro-3*H*-1,2,4-triazole-3-thione (TP-315), after chronic administration to mice. TP-315 was administered to mice intraperitoneally for 14 days. At 24 h post the last injection, animals were decapitated, their brains were acquired, flash-frozen in liquid nitrogen and cut into 10 µm slices. The FT-IR chemical imaging technique was used for the investigation of the potential neurotoxic effect in the cerebral cortex and hippocampus. The effect on the lipidomic and proteomic profile and on oxidative stress was investigated. The results showed no statistically significant changes in the above-mentioned parameters. TP-315 seems to pose no neurotoxic effect on the mouse brain after chronic use, therefore, its use should be safe.

## 1. Introduction

Epilepsy is a chronic neurological disease associated with the presence of neuronal conduction defects and disturbed neuronal excitability [1]. Currently, pharmacotherapy is the main treatment for epilepsy. The drugs used act symptomatically by reducing or completely eliminating the occurrence of epileptic seizures, and do not show a causal effect enabling the elimination of organic changes in the central nervous system [2]. The mechanisms of action of anti-seizure medications are mainly based on the enhancement of GABAergic transmission, the blocking of potential-dependent sodium and calcium ion channels, inhibition of the release of neurotransmitters from synapses and activation of potassium ion channels [3]. Despite the significant progress in research on anti-seizure medications, epilepsy continues to be a serious therapeutic problem, leading to a reduction in the quality of life of patients. The research on new anticonvulsants conducted in recent years has not led to the synthesis of drugs that will be fully effective in monotherapy of various types of epileptic seizures without causing increased side effects. Unfortunately, one of the main reasons for the failure of the applied therapy are the side effects associated with the chronic use of anti-seizure medications. Therefore, a good anti-seizure medication is expected to be effective in reducing the occurrence of seizures in various forms of epilepsy and in effective doses to cause as little side effects as possible during chronic administration [4,5].

In our recent studies, we have shown that 5-(3-chlorophenyl)-4-hexyl-2,4-dihydro- 3*H*-1,2,4-triazole-3-thione (TP-315) is a good candidate for an anti-seizure medication due to its anticonvulsant activity in the MES (maximal electroshock-induced seizure) and the 6 Hz tests (six-hertz seizure test—screening test for the effectiveness of potential drugs in the treatment of drug-resistant epilepsy [6]). During short-term administration of the compound to mice, no acute neurotoxic effect was observed in the rotarod test [7,8]. The mechanism of the compound’s anticonvulsant activity may be related to its effect on voltage-gated sodium channels. TP-315 slightly influenced the viability of HepG2 cells [9]. No hepatotoxic or nephrotoxic effects were observed during chronic administration of the compound to living organisms. TP-315 also did not cause disturbances in morphological parameters. The compound showed high permeability through the blood–brain barrier and did not inhibit selected isoforms of CYP-450 at the concentration determined in the serum of mice during chronic administration [10]. As the compound can cross the blood–brain barrier, it might possess a neurotoxic effect, especially with chronic administration.

The aim of this study was to investigate the potential neurotoxic effect of TP-315 after chronic administration with the use of Fourier-transformed infrared spectroscopic imaging (FT-IR).

## 2. Results and Discussion

### 2.1. Infrared Spectral Analysis

In order to determine the changes in the mouse brain, infrared spectroscopic imaging was performed. The main bands found in the spectra were described in the previous articles and are listed in Table 1 [11,12,13].

The cerebral cortex and the hippocampus were analyzed separately. Neither of the structures presented significant differences in the areas under bands between treated and untreated tissue. However, the cerebral cortex presented higher average differences than the hippocampus (15.24% versus 10.05%, respectively). Infrared spectroscopy of the animal tissue provides valuable information regarding the proteins and lipids. However, it provides only general information about the changes in the carbohydrate types, thus, further analysis was focused on proteins and lipids.

**Figure 1:**
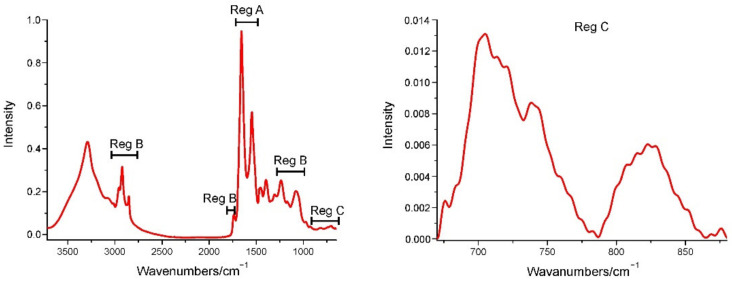
The FT-IR spectra of mouse brain tissue with the regions of interest A, B, C. Region A (Reg A) is the range from 1715 cm^−1^ to 1481 cm^−1^ representing the protein bands, Region B (Reg B) between the range 3032 cm^−1^ to 2810 cm^−1^, 1800 cm^−1^ to 1715 cm^−1^ and 1285 cm^−1^ to 1000 cm^−1^ represents the lipid bands, Region C (reg C) between the range 650 cm^−1^ to 875 cm^−1^ is a region where ν O–O of peroxides and ρ CH_2_ of lipids appears, vital for the oxidative stress analysis.

The average FT-IR spectra of the samples are presented in Figure 1.

#### 2.1.1. Lipidomic Analysis

The main constituents of the brain are lipids (c.a. 50% of the dry weight [14]). They possess many important functions: they are a main part of the cell membranes, serve as a biomessengers, take part in signal transduction and are crucial for the anchoring of the marker protein in the membrane [15]. Moreover, lipids seem to play an important role in brain disorders such as Alzheimer’s disease, Parkinson’s disease, Niemann–Pick diseases, multiple sclerosis, Huntington’s disease and schizophrenia [16]. Because of the fact, that the lipids have a wide variety of functions in the brain, even a small disruption in the balance of the brain lipids may lead to severe effects. That is why it is crucial for the drug not to influence the brain lipids significantly.

**Figure 2:**
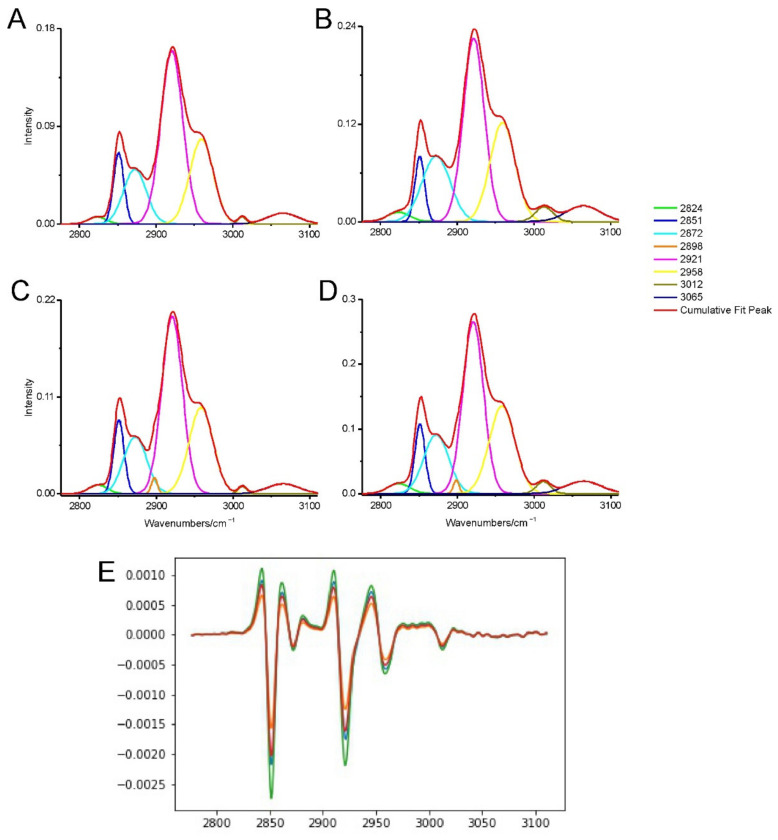
The deconvoluted and second derivative spectra of the lipid region (region B) in the 3110–2800 cm^−1^: (**A**) hippocampus spectra of the control sample. (**B**) hippocampus spectra of the TP-315 treated sample. (**C**) cerebral cortex spectra of the control sample. (**D**) cerebral cortex spectra of the TP-315 treated sample. (**E**) second derivative spectra of control mouse hippocampus (orange spectrum), hippocampus of the treated mouse (blue spectrum), cerebral cortex of the control mouse (red spectrum) and cerebral cortex of the treated mouse (green spectrum). The bands are described in the Table 1.

The spectra of the lipid region are presented in Figure 2. In order to investigate if TP-315 influenced the lipids in a mouse brain, FT-IR spectroscopic imaging was performed. The overall lipid amount (2994–2800 cm^−^^1^ region) in the hippocampus raised slightly (5.29%). In the cerebral cortex, it also raised by 27.03%. The amount of all esters (ν_as_ CO–O–C of esters and ν_s_ CO–O–C of esters) decreased both in the hippocampus and cerebral cortex by 2.7% and 5.83%, respectively. The amount of carbonyl esters (ν C=O of carbonyl ester) corresponded to the amount of phospholipids, which increased (9.94%) in the hippocampus, while it decreased (7.7%) in the cerebral cortex. Moreover, the amount of unsaturated fatty acids increased both in the mouse hippocampus (18.25%) and cerebral cortex (32.63%) treated with the TP-315. The ratio of the proteins to lipids decreased slightly (2.79% and 16.47%) in both the hippocampus and cerebral cortex, respectively. Despite the high percentage change (especially in the amount of unsaturated fatty acids), all of above-mentioned results were not statistically significant, therefore, there were no significant changes in the amount of lipids in the brain.

Moreover, to test, if the membrane phospholipids’ fluidity, packing and ordering changed, the shift analysis was performed. In the hippocampus, the ν_as_ CH_2_ lipid band shifted from 2920.98 (±0.56) cm^−^^1^ to 2921.3 (±0.56) cm^−^^1^, ν_s_ CH_2_ lipid band shifted from 2851.23 (±0) cm^−^^1^ to 2851.55 ± 0.56) cm^−^^1^. Moreover, there were no shifts in the bands corresponding to ν C=O of carbonyl ester, ν_as_ PO_2_^−^ of phospholipids and nucleic acids and ν_as_ and, ν_s_ CO–O–C of esters. In the cerebral cortex, the ν_as_ and ν_s_ CH_2_ of the lipid bands were only slightly shifted (2920.66 (±0) cm^−^^1^ to 2921.14 (±0.56) cm^−^^1^ and 2851.23 (±0) cm^−^^1^ to 2851.47 (±0.48) cm^−^^1^, respectively) and not statistically significant, indicating that the membrane fluidity did not change. Therefore, the second derivative analysis showed no influence on the position of the investigated lipid bands (Figure 2E). Moreover, the bands corresponded to other lipids (mentioned in the hippocampus part) also were not affected by the treatment. The results investigating the change of the fluidity, packing and ordering were statistically insignificant, indicating that TP-315 does not affects these features.

#### 2.1.2. Proteomic Analysis

Proteins are one of the most important constituents both in the brain and the whole body. Due to their ability to form complex structures they possess many functions. In the brain, they serve as transporters, catalysts, receptors, provide a proper cell structure and many more [17,18,19,20]. Abnormalities in the protein structure are present in Alzheimer’s, Parkinson’s and other neurodegenerative diseases [21,22]. Even aging causes alteration in the brain proteins [23]. It is therefore important to investigate the possible influence of the newly invented drug on the protein structure in the brain.

**Figure 3:**
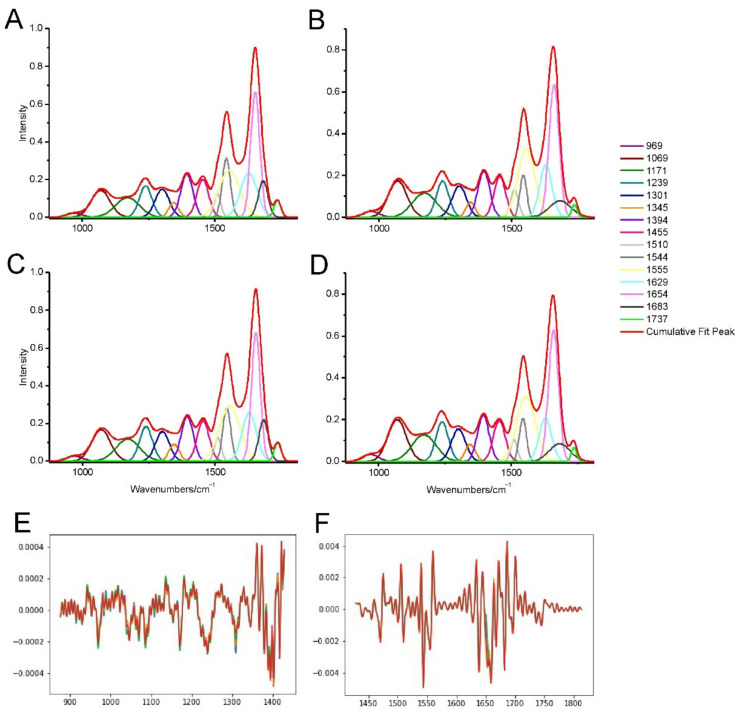
The deconvoluted and second derivative spectra of the protein (region A) and lipid region (region B) in the 1780–900 cm^−1^. The protein region is in the 1717–1485 cm^−1^. The lipid region is split into 1770–1718 cm^−1^ and 1275–996 cm^−1^: (**A**) hippocampus spectra of the control sample. (**B**) hippocampus spectra of the TP-315 treated sample. (**C**) cerebral cortex spectra of the control sample. (**D**) cerebral cortex spectra of the TP-315 treated sample. (**E**) second derivative spectra in the 1428–876 cm^−1^ range of control mouse hippocampus (orange spectrum), hippocampus of the treated mouse (blue spectrum), cerebral cortex of the control mouse (red spectrum) and cerebral cortex of the treated mouse (green spectrum). (**F**) second derivative spectra in the 1814–1428 cm^−1^ range of control mouse hippocampus (orange spectrum), hippocampus of the treated mouse (blue spectrum), cerebral cortex of the control mouse (red spectrum) and cerebral cortex of the treated mouse (green spectrum). The bands are described in Table 1.

The spectra of the protein region are presented in Figure 3. TP-315 presented almost no changes in the amount of the proteins both in the hippocampus and the cerebral cortex (0.6% increase and 1.23% decrease, respectively). The amount of α-helical to β-sheet structure also was only slightly affected by the compound in both of the structures (3.29% increase in the hippocampus and 1.23% decrease in the cerebral cortex). All of above-mentioned changes were, however, statistically insignificant, therefore, TP-315 does not exert an influence on the brain proteins. The second derivative analysis showed no statistically significant shifts in the investigated protein bands (Figure 3E).

#### 2.1.3. Oxidative Stress Analysis

The brain is an organ that is very susceptible to oxidative stress. This is mainly due to the high concentration of unsaturated fatty acids, high amount of oxygen that the brain uses, high amount of iron and ascorbate and low amount of mechanisms that protect the brain from oxidative stress [24]. Oxidative stress also takes part in most brain diseases, namely: Alzheimer’s disease, mild cognitive impairment, Parkinson’s disease, amyotrophic lateral sclerosis and stroke [25]. Moreover, data shows that the excessive amount of oxidative stress leads to the frailty of the organisms [26]. Taking into account, that the anticonvulsant drug crosses the blood–brain barrier, it should possess as little oxidative potential as possible.

**Figure 4:**
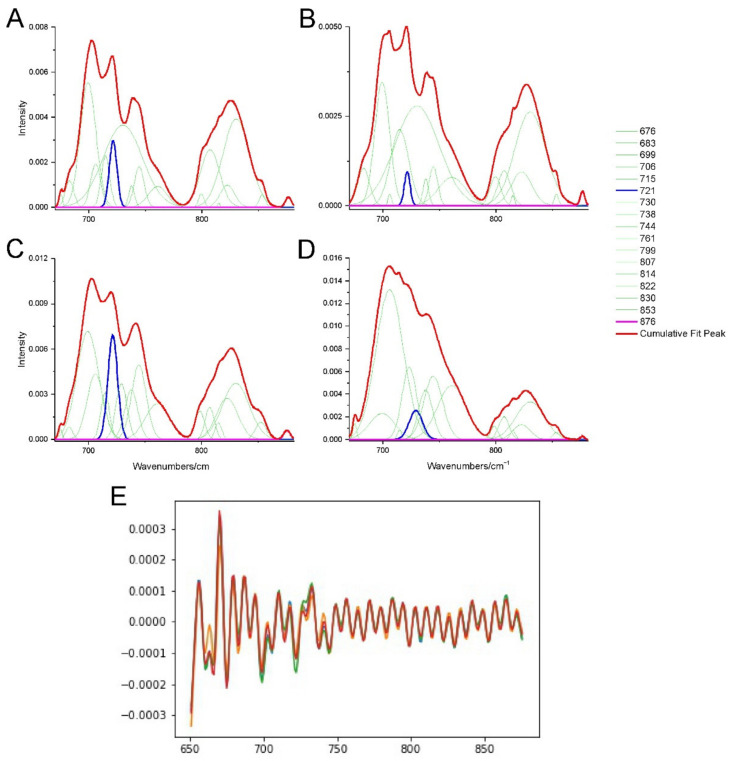
The deconvoluted and second derivative spectra of the oxidative stress region (region C) in the 882–670 cm^−^^1^: (**A**) hippocampus spectra of the control sample. (**B**) hippocampus spectra of the TP-315 treated sample. (**C**) cerebral cortex spectra of the control sample. (**D**) cerebral cortex spectra of the TP-315 treated sample. The interesting bands are marked with blue and pink color. (**E**) second derivative spectra of control mouse hippocampus (orange spectrum), hippocampus of the treated mouse (blue spectrum), cerebral cortex of the control mouse (red spectrum) and cerebral cortex of the treated mouse (green spectrum) The bands are described in Table 1.

The spectra of the oxidative stress region are presented in Figure 4. TP-315 presented quite a high increase in the ratio of the area under the band corresponding to ν O–O of peroxides to the area under the band corresponded to ρ CH_2_ of lipids in the hippocampus (59.57%). In the cerebral cortex it was decreased (3.37%). However, both of these results were statistically insignificant, indicating that these differences are due to the individual variability of the mice and that the compound does not cause increased peroxidation. Moreover, the investigated bands showed no statistically significant band shifts (Figure 4E). This is further supported by previous studies [8].

### 2.2. Infrared Chemical Images Analysis

**Figure 5:**
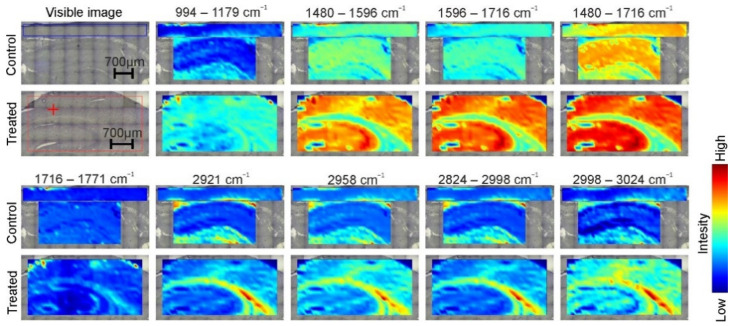
FT-IR images of cortex and hippocampus of the control and anticonvulsant-treated sample. The spatial distributions of selected bands lipids and amides are presented. The bands assignment are described in Table 1.

In order to investigate the distribution of selected metabolites in the brain tissue slices, spectroscopic chemical FT-R imaging was performed (Figure 5). The chemical maps revealed some differences within the spatial distribution of selected bands in the tissues. The band at 994–1179 cm^−^^1^ corresponded to ν CO–O–C of esters and was distributed on the edges of the hippocampus with a higher abundance than in the center of the sample treated with TP-315, while in the control sample this band was distributed evenly throughout the hippocampus. The distribution of the band at 1716–1771 cm^−^^1^ corresponded to ν C=O of carbonyl esters (phospholipids) which also differed. While the hippocampus of the control sample has the band distributed evenly, the treated sample has some places with a higher concentration of the bands. The band at 2921 cm^−^^1^ (corresponded to ν_as_ CH_2_ of lipids), at 2958 cm^−^^1^ (corresponded to ν_as_ CH_3_ of lipids), at 2824–2998 cm^−^^1^ (corresponded to CH vibrations of all lipids) and at 2998–3024 cm^−^^1^ (corresponded to ν = C–H of unsaturated lipids) differed between the control and treated sample in the cortex. They differed in a similar manner; therefore, they are described as combinatory. In the control sample, these bands are distributed mainly in the upper edge of the cortex. In the treated sample, however, these bands are concentrated in the specific places of the cortex, mainly in the center. Moreover, the distribution of the band at 2998–3024 cm^−^^1^ also differs in the hippocampus. In the control sample, this band has a slight increase in the intensity in the center, while in the treated sample it is distributed evenly throughout the structure except for the narrow line at some distance from the edge. These results suggest that the main differences in the distribution were presented in the case of the lipids. The distribution of the proteins was not affected by TP-315 administration and all the differences were minor.

## 3. Materials and Methods

### 3.1. Synthesis of 5-(3-Chlorophenyl)-4-Hexyl-2,4-Dihydro-3h-1,2,4-Triazole-3-Thione (TP-315)

5-(3-chlorophenyl)-4-hexyl-2,4-dihydro-3*H*-1,2,4-triazole-3-thione was synthesized as described in the published article [7]. Reagents necessary for the synthesis were purchased from POCh Gliwice (Gliwice, Poland) and Sigma-Aldrich (St. Louis, MO, USA).

### 3.2. Experiments on Adult Male Albino Swiss Mice

The consent to conduct experiments with the use of Albino Swiss mice was issued by the Local Ethical Committee for Animal Experiments in Lublin (Resolution No. 71/2019). The experiments were performed in the certified Center of Experimental Medicine of the Medical University of Lublin. The experimental group (*n* = 3) was given TP-315 with an ED_50_ dose (47.6 mg/kg body mass) [27] intraperitoneally for 14 days, while the control group (*n* = 3) was treated the same way, but using the saline solution. At 24 h post the last injection, animals were decapitated, their brains were acquired and flash-frozen in liquid nitrogen, then kept at −80 °C.

### 3.3. Sample Preparation and Infrared Spectroscopic Data Acquisition

Mouse brain tissues were sectioned on 10 µm slices using cryomicrotome (Leica CM 1950, Leica Biosystems, Wetzlar, Germany) in the frontal plane in order to obtain the cortex and hippocampus on one slice. The samples were thaw mounted on aluminum coated slides. The slides were stored at −20 °C. Directly before analysis, the samples were thawed and air-dried. Chemical maps were obtained using Nicolet 6700 FT-IR spectrometer (Thermo Scientific, Waltham, MA, USA) in transflection mode with a step size of 100 µm × 100 µm on the *x* and *y* axis. The aperture used was 100 µm × 100 µm. The spectral resolution was 8 cm^−1^. The objective magnification used was 15×.

### 3.4. Data Preprocessing and Semi-Quantitative Analysis

Baseline correction, deconvolution and spectra analysis were performed using Origin Pro Software (v. 9.1, OriginLab Corporation, Northampton, MA, USA). Chemical map analysis was performed using CytoSpec Software (v. 2.00.01, Berlin, Germany). The average differences were calculated as follows: the area from each band of the specific region (cortex or hippocampus) was averaged in each group (control and treated). The percentage of differences between the treated and control group was calculated. The mean difference was then presented. Then all the differences from each region were averaged and it resulted in average differences of the region. The overall protein amount was calculated by the sum of the area under Amide I and Amide II band. The overall lipid amount was calculated by the sum of all the areas under lipid bands in the 2994–2800 cm^−^^1^ region.

### 3.5. Statistical Analysis

Statistical analysis of the areas under the bands was performed by one-way analysis of variance (ANOVA) with Tukey’s post hoc test in the Statistica 13 software (v. 13.3. TIBCO Software Inc., Palo Alto, CA, USA). The statistically significant data were considered to have *p* ≤ 0.05. Three average spectra from the specific brain region (*n* = 3) were used for the statistical analysis.

### 3.6. Protein Structural Analysis

The changes in protein structures were calculated by the ratio of the area under α-helix assigned bands at the ~1654 cm^−^^1^ to β-sheet assigned band at ~1628 cm^−^^1^ and ~1683 cm^−^^1^ (Table 1) (Figure 1 Reg A).

### 3.7. Lipidomic Structural Analysis

The changes in the lipidomics were calculated by the ratio of the area under a specific lipid band to the area of the wide band of the lipids (2994–2800 cm^−^^1^). The change of the fluidity, packing and ordering of the membrane phospholipids in the brain was investigated using shifts of the band center corresponding to lipids (ν_as_ and ν_s_ CH_2_ of lipids, ν C=O of carbonyl ester, ν_as_ PO_2_^−^ of phospholipids and nucleic acids, ν_as_ and, ν_s_ CO–O–C of esters (Table 1)). The change of membrane fluidity and the length of the lipids was also calculated by the change of the ratio of the area of ν_as_ CH_2_ of lipids band to ν_as_ CH_3_ of the lipids band (Table 1) (Figure 1 Reg B) [28].

### 3.8. Oxidative Stress Analysis

The level of oxidative stress in the selected areas of the brain was measured by calculating the ratio of the area under the band corresponding to ν O–O of peroxides to the area under the band corresponded to ρ CH_2_ of lipids (Table 1) (Figure 1 Reg C) [11,13].

## 4. Conclusions

In this study, the potential neurotoxic effect of the TP-315 was investigated with the use of FT-IR spectroscopic imaging. The effect on the amount of lipids, their structural change and distribution, the effect on the amount of proteins and their secondary structures and their distribution and the effect on the oxidative stress were investigated. All of these features showed some differences, but all of them were statistically insignificant, therefore proving that TP-315 does not have neurotoxic potential during chronic administration to living organisms.

## Figures and Tables

**Table 1:** Bands found in the mouse brain spectra with their assigned components.

Wavenumbers [cm^−1^]	Assignment and Type of Vibration
~3012	ν = C–H of unsaturated lipids
~2958	νas CH_3_ of lipids
~2921	νas CH_2_ of lipids
~2900	ν CH of carbohydrates
~2872	νs CH_3_ of lipids
~2851	νs CH_2_ of lipids
~1735	ν C=O of carbonyl ester
~1683	Antiparallel β-sheet structure of proteins (Amide I band)
~1654	α-helix structure of proteins (Amide I band)
~1628	Antiparallel β-sheet structure of proteins (Amide I band)
~1510, ~1544 and ~1555	Multiple bands of Amide II
1455–1300	σ and ρ CH and OH of carbohydrates (numerous bands)
~1239	νas PO2- of phospholipids and nucleic acids
~1171	νas CO–O–C of esters
~1069	νs CO–O–C of esters
~970	σ pyranose ring of carbohydrates
~873	ν O–O of peroxides
~720	ρ CH_2_ of lipids

## Data Availability

The data that support the findings of this study are available from the corresponding author upon reasonable request.
